# Evaluation of carotid artery elasticity changes in patients with type 2 diabetes

**DOI:** 10.1186/1475-2840-13-39

**Published:** 2014-02-10

**Authors:** Li Zhang, Ji-Kai Yin, Yun-You Duan, Xi Liu, Lei Xu, Jia Wang, Yi-Lin Yang, Li-Jun Yuan, Tie-Sheng Cao

**Affiliations:** 1 Department of Ultrasound Diagnosis, Tangdu Hospital, Fourth Military Medical University, Xi’an, China; 2 Department of General Surgery, Tangdu Hospital, Fourth Military Medical University, Xi’an, China

**Keywords:** Arterial stiffness, Intima-media thickness, Diabetes, Strain

## Abstract

**Background:**

Type 2 diabetes is one of the most common causes of cardiovascular disease as it causes arterial stiffness changes. The purpose of this study is to characterize, in vivo, carotid arterial structural and functional changes by applying radio frequency and X-strain ultrasound techniques.

**Methods:**

Ninety-one subjects were assigned into two groups; a diabetes group and a control group. Structural and functional changes in the common carotid arterial wall were investigated by quality intima-media thickness (QIMT), quality arterial stiffness (QAS), and X-strain analysis with a Mylab Twice ultrasound instrument. The relationships among variables between the two groups were analyzed in this study.

**Results:**

There was no significant difference in carotid IMT (626.5 ± 169.1 μm vs. 568.5 ± 122.6 μm, *P* = 0.1506) between two groups. Pulse wave velocity (PWV) and stiffness index (β) were remarkably greater (8.388 ± 3.254 m/s vs. 7.269 ± 1.332 m/s; 12.51 ± 14.16 vs.9.279 ± 2.871), while compliance coefficient (CC) decreased significantly in the diabetes group (0.802 ± 0.3094 mm^2^/Kpa vs. 0.968 ± 0.3992 mm^2^/Kpa) (*P* < 0.05). The displacement difference of radial (RD-D), longitudinal (LD-D) and rotation (ROT-D) directions were significantly different between two groups’ comparison (*P* = 0.0212, *P* = 0.0235 and *P* = 0.0072, respectively). The time of circumferential peak strain difference (CS-DT) and the time of radial peak strain rate (RSR-T) were found to be significantly different between the two groups (341.9 ± 77.56 ms vs. 369.0 ± 78.26 ms, *P* = 0.0494; 142.7 ± 22.43 ms vs. 136.2 ± 30.70 ms, *P* = 0.0474). CS-TD and RSR-T were also found to be positively correlated with CC value (r = 0.3908, *P* < 0.005 and r = 0.3027, *P* = 0.0326, respectively). Finally, PWV was negatively correlated with CC with (r = –0.6177, *P* < 0.001).

**Conclusions:**

In type 2 diabetes, the functional changes in CCA can be identified using the methods presented in this article earlier than the structural changes. Arterial stiffness values provided by QAS and X-strain analysis can be used as indicators of CCA functional lesions in patients with type 2 diabetes.

## Background

Diabetes is a major contributor to atherosclerosis of the arterial bed [1,2]. Patients with diabetes are at a high risk of artery atherosclerosis leading to cardiovascular disease (CVD), especially coronary heart disease (CHD), which is the most common complication and the principal cause of death in type 2 diabetes patients. The carotid artery can be considered as a model to reflect conditions common to all infected arteries. Detection of structural and functional disorders of the common carotid artery (CCA) by duplex ultrasonography is most favorite method in evaluating systemic artery atherosclerosis.

CCA intima-media thickness (IMT) measurements are considered a strong predictor of future vascular events and a surrogate marker of atherosclerosis [3-5], although there are some recent studies that contest this theory [6,7]. Arterial elasticity assessment is also regarded as an independent predictor of cardiovascular mortality and morbidity in patients with cardiovascular disease as well as in healthy individuals [8,9].

Ultrasound radio frequency (RF) technology is a newly developed modality used for evaluating vascular elasticity of the carotid artery; the measurement of artery elasticity is based on the monitoring of RF signals transmitted by ultrasound. Data acquired by this technology can reflect changes objectively and accurately both from structural and functional aspects. X-strain technology, a vector strain imaging-VSI™, based on angle independent speckle-tracking technology applied to myocardial strain measurements to reduce error compared to Doppler tissue imaging [10,11]. In the present study, we adapt this method for carotid arterial stiffness analysis aiming to assess functional artery changes in patients with type 2 diabetes. In addition, we discuss change tendencies in the carotid artery and how they are identified using this superior technology.

## Methods

### Ethical approval of the study protocol

All subjects included in the study provided written informed consent. The study protocol was approved by the ethics committee of the Fourth Military Medical University Tangdu Hospital (Xi’an, China).

### Patient selection

Between March 2012 and September 2012, 50 patients diagnosed with type 2 diabetes (treatment with diet an oral drugs) by laboratory tests at Tangdu Hospital of the Fourth Military Medical University, were investigated in this study. Patients with hypertension, hyperlipidemia, coronary heart disease, and nephropathy were excluded from the study. In order to avoid errors derived from subject matching, 41 healthy volunteers (22 males and 19 females; age, 40–79 years) were matched with patients based on gender and age. Before ultrasound measurement, we collected data from the physical examination and laboratory tests of each study subject. The following laboratory parameters were obtained: total cholesterol (TC), low density lipoprotein (LDL), high density lipoprotein (HDL), triglycerides (TG), HbA1c, serum creatinine, uric acid and HAb1c. Other clinical data includes clinical blood pressure determined by performing three systolic blood pressure tests (SBP), and diastolic blood pressure (DBP) measurements, body mass index (BMA) and body surface area (BSA).

### Ultrasound examination

Ultrasound examinations were conducted with a Mylab Twice color Doppler ultrasound diagnostic system (Esaote, Firenze, Italy), using a 5-13 MHz vascular probe LA523 with built-in quality intima-media thickness (QIMT), quality arterial stiffness (QAS), and X-strain analysis software. After each subject was placed in the supine position, the right common carotid artery (RCCA), carotid bulb, and portions of the internal carotid arteries on both sides were scanned. The region of interest (ROI) was defined as 30 mm proximal to the beginning of the dilation of the bifurcation bulb.

The CCA examination was performed by two ultrasound physicians with 10 years working experience who had received formal training in vascular screening. The physicians were blinded to any clinical information regarding the subjects.

### QIMT analysis

**Figure 1:**
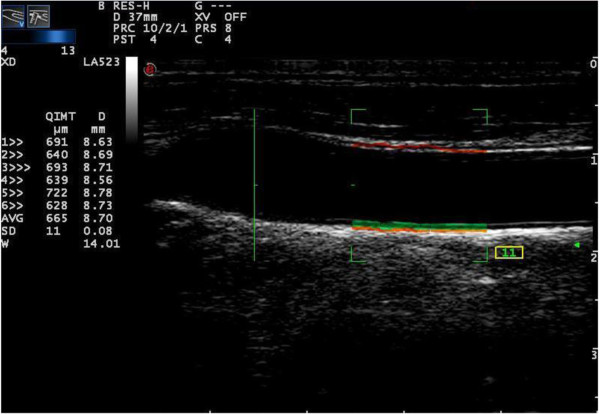
**QIMT analysis of the common carotid artery.** The red line represents the radiofrequency signal tracking the leading edge of the lumen intima; the green line represents the radiofrequency signal tracking the leading edge of media adventitia interface. The IMT value and vascular diameter was calculated automatically for six cardiac cycles showed on the left side of the picture.

After the subject was placed in the correct position, the CCA was showed in a longitudinal view. The ultrasonographic image was focused on the QIMT measurement site and ensured that the anterior and posterior walls of the CCA were clearly shown. The regions with plaques were avoided in the ROI of CCA. By pressing the “Tools” button and starting the QIMT function, a RF signal tracked the leading edge of lumen intima to the leading edge of media adventitia interface at the posterior wall of the selected vascular segment. The software automatically acquired six cardiac cycle QIMT measurements (Figure 1). When the standard deviation (SD) was less than 15, the image was frozen and stored for further analysis.

### QAS analysis

**Figure 2:**
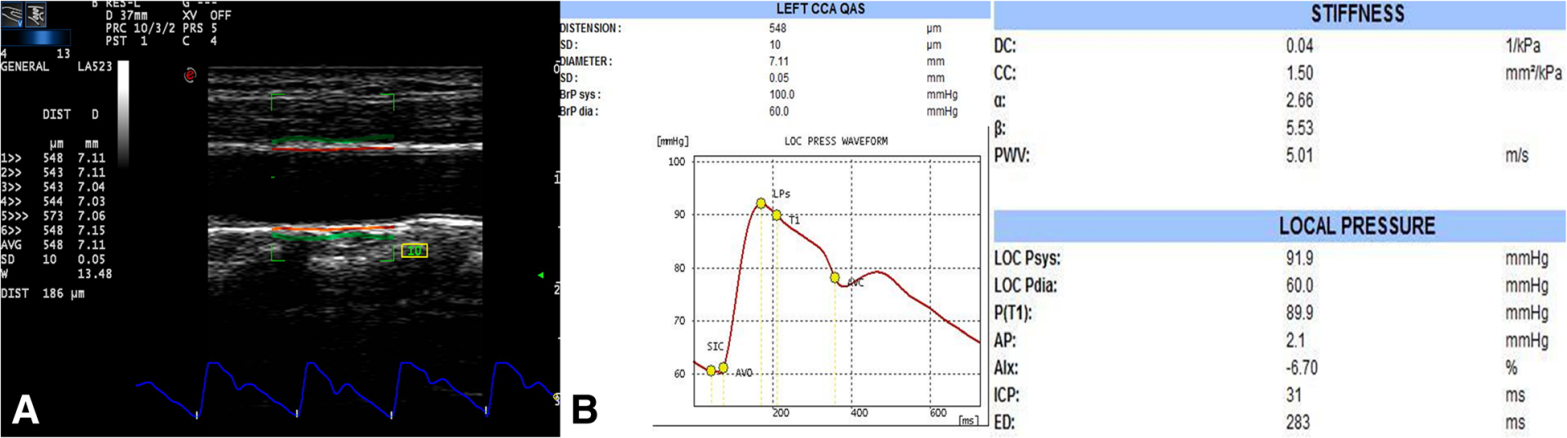
**QAS analysis of the common carotid artery. (A)** The red line represents the radiofrequency signal tracking the leading edge of the lumen intima; the green line represents the radiofrequency signal tracking the leading edge of media adventitia interface. **(B)** The stiffness value was calculated automatically for six cardiac cycles.

Concurrently with the QIMT measurement, QAS measurements began after pressing the “Tools” button. An RF signal tracked the vascular wall while another signal tracked the motion of vascular wall for at least for six cardiac cycles and calculated the mean and SD values automatically. SD value was controlled under a cutoff value 15 (Figure 2A). QAS data analysis software also calculated the pulse wave velocity (PWV), compliance coefficient (CC), and stiffness index (αand β; Figure 2B).

### X-strain analysis

**Figure 3:**
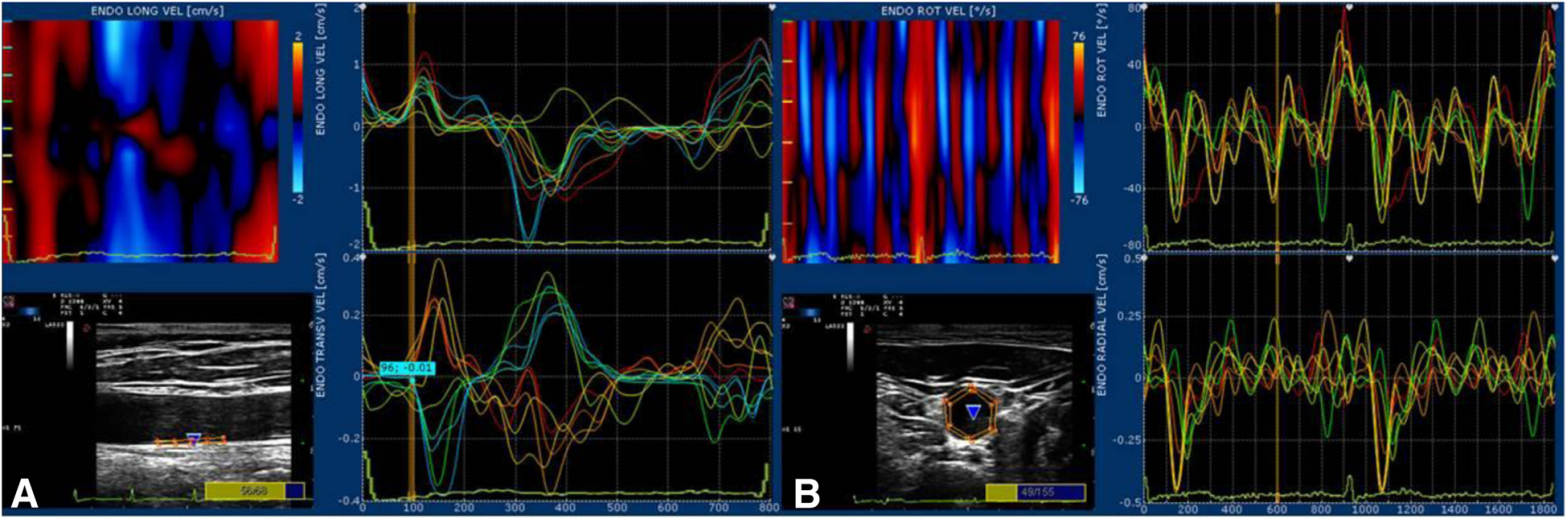
**X-strain analysis of the common carotid artery.** Longitudinal **(A)** and transverse **(B)** views were shown in the same position of the common carotid artery. The motion of the lumen intima and the adventitia were colorized as shown in the upper side of left column. The curves of different variables by X-strain analysis were traced automatically (the right column). In the pictures, we provide velocity curves analyzed by the X-strain method.

The X-strain analysis site is located 1 cm proximal to the bifurcation. Long and short axis were showed in the same measurement site The sampling sites were placed at the leading edge of the lumen intima and the leading edge of media adventitia interface at the posterior wall. The RF signal tracked both the motion of vascular intima and adventitia for at least three consecutive heart beats. The real-time images were stored and offline analysis was performed using a workstation equipped with Mylab desk analysis software (Figure 3). Depending on the view under analysis the following parameters were calculated as shown in Table 1.

**Table 1:** Parameters of different views of X-train analysis

**Parameters of long axis view**	**Unit**
Longitudinal displacement	mm
Longitudinal strain	%
Radial strain	%
Longitudinal strain rate	1/sec
Radial strain rate	1/sec
**Parameters of short axis view**	**Unit**
Rotational displacement	Degree
Radial displacement	mm
Circumferential strain	%
Radial strain	%
Circumferential strain rate	1/sec
Radial strain rate	1/sec

Except that the radial strain and strain rate was output as the difference of the intima and adventitia values by the software, other parameters were output as the intima and adventitia values respectively. So variables are expressed as D (the intima and adventitia peak value difference), DT (the time of the intima and adventitia peak value difference) and TD (the time difference of the intima and adventitia peak value). Significant difference between the two group and correlations among these variables were evaluated.

### Statistical analyses

All data are expressed mean ± SD. Differences between the two groups were tested using unpaired Student *t*-tests. The reproducibility of the arterial stiffness measurements was tested in the younger subjects and the intra- and inter-observer variability was assessed by linear correlation analysis and Bland-Altman plots. Linear regression analysis was used to assess the correlations among all parameters. Results were considered significant at *P* < 0.05. The statistical software package SPSS 12.0 (IBM Corporation, Armonk, NY, USA) was used for all data analyses.

## Results

### Patient characteristics

The main clinical and pathological data for patients at the beginning of the study are presented in Table 2. BSA, DBP, SBP, total cholesterol, LDL cholesterol, TC and HAb1c were higher in patients with diabetes. There were no significant differences between groups with respect to the other characteristics concerned.

**Table 2:** Clinical characteristics of the study subjects

**Characteristics**	**Controls**	**Patients**	**P value**
Gender (M/F)	19/22	22/28	
Age (mean ± SD)	53.05 ± 11.99	53.60 ± 9.29	0.814
Height (cm)	164.80 ± 7.49	16 6.06 ± 8.26	0.471
Weight (kg)	67.15 ± 8.10	70.45 ± 17.98	0.290
BMI (kg/m^2^)	24.68 ± 2.01	25.49 ± 5.83	0.407
BSA (m^2^)	1.667 ± 0.1746	1.754 ± 0.1778	0.0257*
SBP (mmHg)	116.11 ± 12.18	134.9 ± 18.81	0.0007*
DBP (mmHg)	79.84 ± 9.241	86.50 ± 12.09	0.0036*
Duration of diabetes (years)	/	5.47 ± 2.37	/
Smoker/duration (%/years)	32%/8.23 ± 6.74	36%/9.72 ± 4.43	0.379
TC (mmol/l)	3.82 ± 0.31	4.42 ± 0.12	<0.0001*
HDL cholesterol (mmol/l)	1.63 ± 0.13	1.58 ± 0.17	0.137
LDL cholesterol (mmol/l)	2.94 ± 0.24	3.12 ± 0.08	0.036*
TG (mmol/l)	1.02 ± 0.65	1.13 ± 0.87	0.236
HbA1c (%)	4.38 ± 1.43	7.52 ± 1.05	<0.0001*
Serum creatinine (μmol/l)	47.82 ± 12.5	50.31 ± 26.7	0.098
Uric acid (μmol/l)	221.43 ± 25.96	229.57 ± 23.2	0.569

**Notes:** Data are presented as means ± standard deviation. *Value between controls and patients with diabetes *P* < 0.05.

*Abbreviations:BMI* body mass index; *BSA* body surface area; *SBP* systolic blood pressure; *DBP* diastolic blood; *TC* total cholesterol; *HDL* high density lipoprotein; *LDL* low density lipoprotein; *TG* triglycerides.

### Arterial structure and stiffness detection

Carotid arterial plaque was found in 16% of the control group and 37% of the patient group, which was not significantly different between patients group and control group. No significant alteration in IMT was seen in the patient group compared to controls (*P* = 0.1506). The results of QAS analysis in Table 3 shows that PWV and β arterial stiffness parameters were much higher in the patient group, while CC was significantly lower (*P* = 0.0430, *P* = 0.0463, and *P* = 0.0414 respectively). The stiffness index αshowed a little higher in patients group, while no significant difference was found.

**Table 3:** Comparison between the two groups by QIMT and QAS measurements

**Variables**	**Patients**	**Controls**	**P value**
IMT (μm)	626.5 ± 169.1	568.5 ± 122.6	0.1506
PWV (m/s)	8.388 ± 3.254	7.269 ± 1.332	0.0430*
α	5.481 ± 7.208	4.987 ± 1.749	0.1429
β	12.51 ± 14.16	9.279 ± 2.871	0.0463*
CC (mm^2^/Kpa)	0.802 ± 0.3094	0.968 ± 0.3992	0.0414*

**Notes:** *Value between controls and patients with diabetes *P* < 0.05. Data are presented as means ± standard deviation.

*Abbreviations:IMT* intima-media thickness; *PWV* pulse wave velocity; *CC* compliance coefficient.

### X-strain analysis

The results of the X-strain analysis indicate that radial displacement difference (RD-D) and longitudinal displacement difference (LD-D) were higher in diabetes patients compared to the control group, while rotation displacement difference (ROT-D) decreased in the patient group (*P* = 0.0212, *P* = 0.0235 and *P* = 0.0072, respectively) (Table 4). In addition, the time difference of radial peak displacement (RD-TD) and the time of rotation peak displacement difference (ROT-DT) in the patient group presented earlier than in the healthy controls. No significant differences were found between the two groups as far as other variables were concerned.

**Table 4:** Comparison of displacement variables between the two groups by X-strain analysis

**Variables**	**Patients group**	**Control group**	**P value**
**LD-D (mm)**	0.0884 ± 0.053	0.0638 ± 0.028	0.0212*
**LD-DT (ms)**	390.0 ± 163.1	365.1 ± 205.1	0.2262
**LD-TD (ms)**	35.96 ± 27.78	65.14 ± 67.36	0.0420*
**RD-D (mm)**	0.8188 ± 0.5993	0.6000 ± 0.2290	0.0235*
**RD-DT (ms)**	314.7 ± 87.15	337.3 ± 79.16	0.1074
**RD-TD (ms)**	137.8 ± 49.7	130.8 ± 43.89	0.5080
**ROT-D (mm)**	0.4981 ± 0.2225	0.6876 ± 0.3874	0.0161*
**ROT-DT (ms)**	316.4 ± 79.9	394.3 ± 147.1	0.0072*
**ROT-TD (ms)**	123.7 ± 59.81	122.2 ± 77.56	0.2568

**Notes:** *Value between controls and patients with diabetes *P* < 0.05. Data are presented as means ± standard deviation.

*Abbreviations:RD-D* radial displacement difference; *RD-DT* the time of radial peak displacement difference; *RD-TD* the time difference of radial peak displacement; *LD-D* longitudinal displacement difference; *LD-DT* the time of longitudinal peak displacement difference; *LD-TD* the time difference of longitudinal peak displacement; *ROT-D* difference of rotation displacement; *ROT-DT* the time of rotation peak displacement difference; *ROT-TD* the time difference of rotation peak displacement.

With respect to the type of strain and strain rate variables, only the time of circumferential peak strain difference (CS-DT) and the time of radial peak strain rate (RSR-T) were found to be significantly different between the two groups (341.9 ± 77.56 ms vs. 369.0 ± 78.26 ms, *P* = 0.0494; 142.7 ± 22.43 ms vs. 136.2 ± 30.70 ms, *P* = 0.0474) (Table 5 and Table 6). Other variables showed no obviously different between two groups.

**Table 5:** Comparison of strain variables between the two groups by X-strain analysis

**Variables**	**Patients group**	**Control group**	**P value**
**LS-D (%)**	1.936 ± 0.9507	1.601 ± 0.5719	0.1136
**LS-DT (ms)**	359.5 ± 78.11	379.2 ± 138.7	0.7695
**LS-TD (ms)**	65.86 ± 49.97	61.64 ± 36.65	0.9615
**CS-D (%)**	22.51 ± 7.056	21.72 ± 7.576	0.5789
**CS-DT (ms)**	341.9 ± 77.56	369.0 ± 78.26	0.0494*
**CS-TD (ms)**	132.6 ± 52.33	151.3 ± 72.17	0.2715
**RS (%)**	3.191 ± 1.212	3.318 ± 1.586	0.6541
**RS-T (ms)**	276.6 ± 45.37	268.4 ± 62.3	0.3850

**Notes:** *Value between controls and patients with diabetes *P* < 0.05. Data are presented as means ± standard deviation.

*Abbreviations:CS-D* circumferential strain difference; *CS-DT* the time of circumferential peak strain difference; *CS-TD* the time difference of circumferential peak strain; *LS-D* longitudinal strain difference; *LS-DT* the time of longitudinal peak strain difference; *LS-TD* the time difference of longitudinal peak strain; *RS* radial strain; *RS-T* the time of radial peak strain.

**Table 6:** Comparison of strain rate variables between the two groups by X-strain analysis

**Variables**	**Patients group**	**Control group**	**P value**
**CSR-D (1/s)**	0.2433 ± 0.7987	0.2517 ± 0.0968	0.7458
**CSR-DT (ms)**	264.3 ± 115.9	279.7 ± 104.5	0.3555
**CSR-TD (ms)**	71.72 ± 78.20	62.17 ± 76.90	0.1732
**LSR-D (1/s)**	5.761 ± 4.456	6.099 ± 3.517	0.5579
**LSR-DT (ms)**	339.1 ± 147.2	317.6 ± 110.7	0.7933
**LSR-TD (ms)**	155.9 ± 71.13	162.0 ± 78.33	0.9588
**RSR (1/s)**	0.4126 ± 0.1615	1.804 ± 6.997	0.7827
**RSR-T (ms)**	142.7 ± 22.43	136.2 ± 30.70	0.0474*

**Notes:** *Value between controls and patients with diabetes *P* < 0.05.

*Abbreviations:CSR-D* circumferential strain rate difference; *CSR-DT* the time of circumferential peak strain rate difference; *CS-TD* the time difference of circumferential peak strain rate; *LSR-D* longitudinal strain rate difference; *LSR-DT* the time of longitudinal peak strain rate difference; *LSR-TD* the time difference of longitudinal peak strain rate; *RSR* radial strain rate; *RSR-T* the time of radial peak strain rate.

### Correlation of arterial stiffness parameters with X-strain parameters

**Figure 4:**
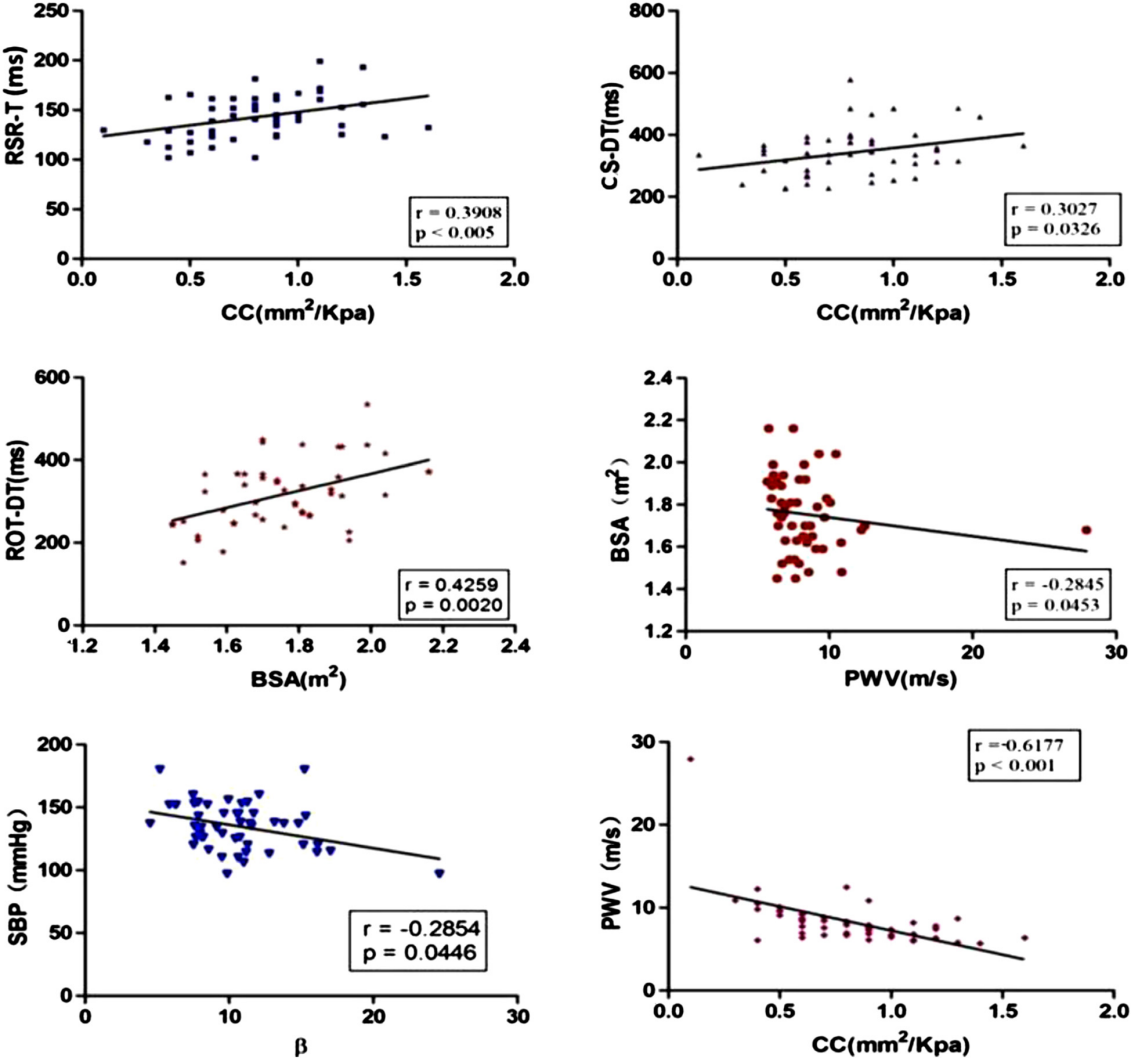
**Correlations of significantly different parameters in the patient group.** Among them, the correlations of BSA and ROT-DT, BSA and β, BSA and PWV, CS-DT and CC, CC and RSR-T, and CC and PWV had statistical significance as shown.

Among the significantly different parameters by X-strain analysis (Figure 4), RSR-T and CS-DT were found to be positively correlated with CC value (*r* = 0.3098, *P* < 0.005 and *r* = 0.3027, *P* = 0.0326, respectively). ROT-DT showed a positive relationship with BSA (*r* = 0.4259, *P* = 0.0020). With respect to the other stiffness and clinical variables, BSA and PWV (*r* = –0.2845, *P* = 0.0453), SBP and β (*r* = –0.2854, *P* = 0.0446), and CC and PWV (*r* = –0.6177, *P* < 0.001) were also found to be negatively associated with each other. No more statistical significance was found among other evaluated variables. Conversely, the significant relationships identified in the patient group were not observed in the control group.

### Repeatability comparison

**Figure 5:**
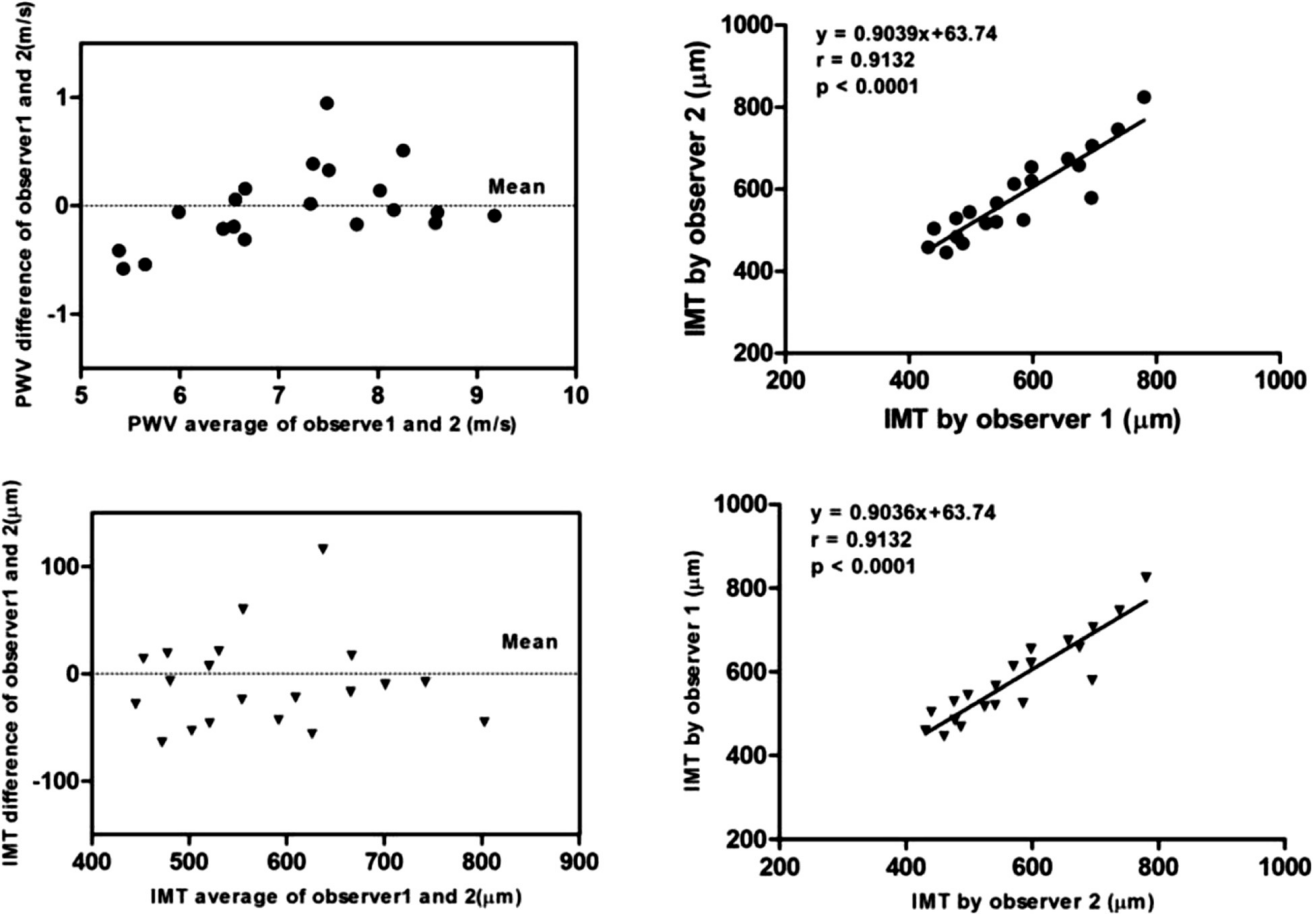
**Inter-observer variability of PWV and IMT measurements.** Linear regression analysis shows good agreement between measurements for PWV and IMT by two independent observers.

**Figure 6:**
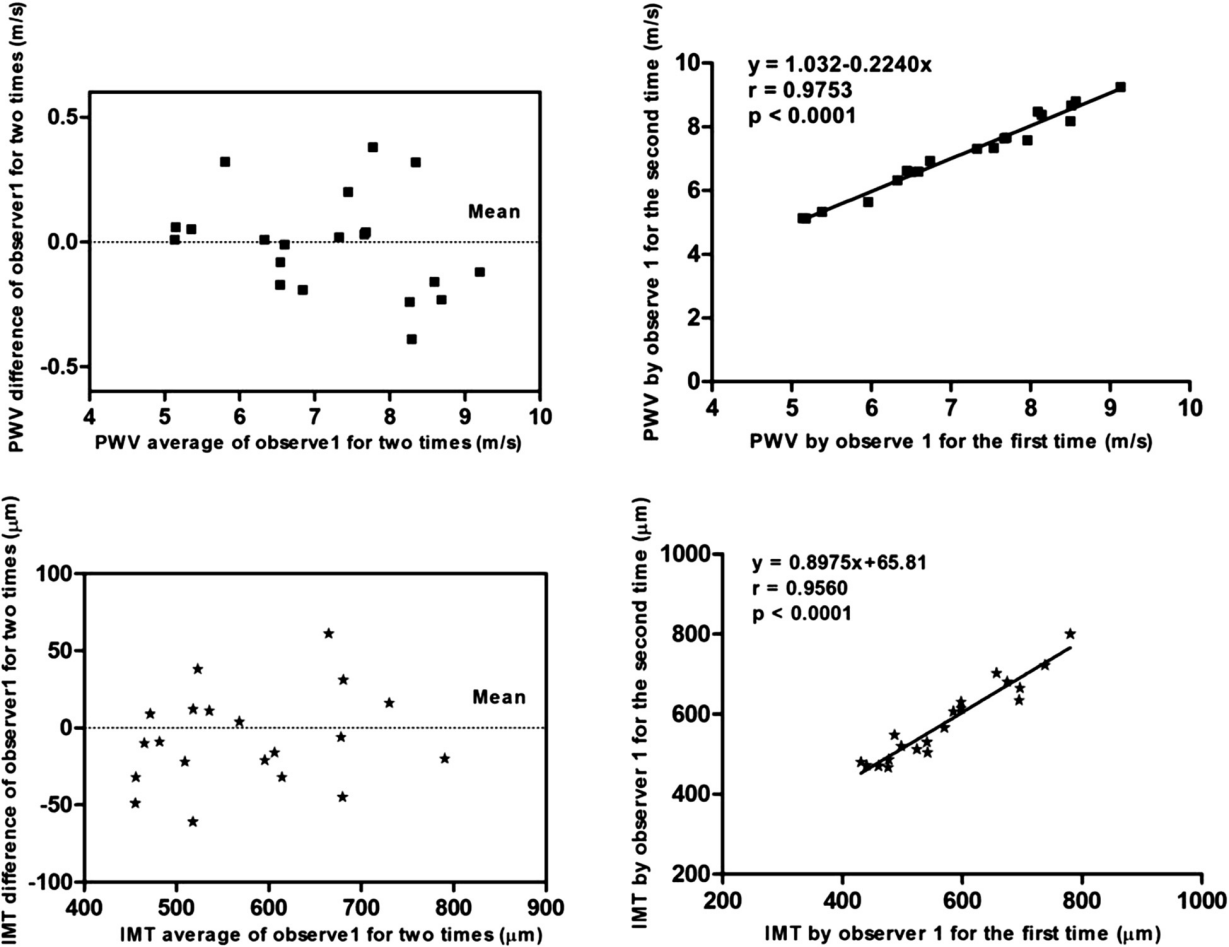
**Intra-observer repeatability of PWV and IMT measurements.** Linear regression analysis shows good agreement between measurements for PWV and IMT by the same observer.

Good agreement was found between intragroup and intergroup comparisons for IMT and PWV values (intragroup: a mean bias of 8.45 ± 43.13 μm; intergroup: a mean bias of 7.05 ± 30.05 μm for IMT; intragroup: a mean bias of 0.013 ± 0.364 m/s; intergroup: a mean bias of 0.007 ± 0.201 m/s for PWV). Bland-Altman analysis showed a consistent trend in the difference and mean values of IMT and PWV by repeated measurement (Figures 5 and 6).

## Discussion

Changes in both structural and functional aspects of arteries have been a research interest for several years as they are considered risk factors for cardiovascular events [12-14]. As diabetes is one of the most common causes of cardiovascular disease, aortic elasticity was impaired in those with impaired fasting glucose [15], in the present study we focused on carotid arterial wall changes induced by type 2 diabetes by evaluating artery lesions using a new method called ultrasound RF data and X-strain technology. By using QIMT and QAS techniques, high resolution ultrasound acquisitions based on RF signals allow us to assess local IMT and stiffness in a rapid and specific manner.

The structural indicators IMT and plaque have been endpoint markers in clinical trials [16-18]. We discovered that IMT of the RCCA showed no obvious changes in patients with diabetes, whereas some of the elastic parameters showed significant differences in these patients relative to controls. Although our findings differed from other reports [4,19], it may be one that supports the notion that functional impairment of the arterial wall may occur early in the atherosclerotic process [20], and arterial stiffening may be a process independent of arterial thickening [21].

Many researches showed to us that arterial stiffness is correlated with the presence and severity of arterial atherosclerosis, and also associated with myocardial dysfunction [22-24]. For functional changes, we assessed both the performance of QAS measurements of vascular stiffness, such as PWV, CC, α, β, and the performance of ultrasonographic speckle tracking-based X-strain measurements. As the most useful and robust index of arterial stiffness [25-27], PWV measured by QAS likely provides accurate local characteristics of vascular alterations. The local vascular stiffness values PWV and β we measured increased in the patient group, while CC decreased, which validates the hypothesis that arterial remodeling occurring in local and elastic arteries increases the risk for future cardiovascular disease.

Besides the stiffness values acquired by QAS, X-strain techniques, a novel method based on the two-dimensional information obtained directly from the arterial wall itself both for the intima and adventitia at the same time, can give us more information about local arterial stiffness, rather than depending on luminal changes. The quantitative assessment of the regional function of the arterial wall may be based on the mobility of the intima, of the adventitia and on wall thickening. Strain and strain rate imaging provides a quantitative method for assessing the regional function of the arterial wall. The variables include the displacement, strain, and strain rate of the arterial wall movement for circumferential, longitudinal, radial, and rotation movements. Like myocardial movement, the intima and adventitial wall stretch in different sectors at the same time can be showed to us by the real-time X-strain analysis. The final arterial wall movement at that moment is decided by the multi-directional forces on both intima and adventitial wall. Unlike other strain analysis technology, X-strain analysis allows us to evaluate all of the forces at the same time and helps us to comprehensively understand the movement of the arterial wall. To our knowledge, this is the first report in the literature to assess arterial stiffness from multiple angles using the speckle tracking method.

Among all these parameters, circumferential, radial, and rotation variables (CS-DT, RSR-T, and ROT-DT) appeared to be sensitive to CCA stiffness changes compared to the longitudinal variables. In mechanics, Laplace’s law and Poisson’s ratio emphasize that when the force on a circumferential or radial section is two times the force on the longitudinal section, the vascular stretching longitudinally can be neglected, suggesting that the movement of an elastic artery is mainly a type of stretching in the circumferential and radial directions and, as such, the movement of the vascular wall is a vascular diameter change, rather than the vascular length change. Therefore, circumferential and radial strain analysis is capable of accurately reflecting local vascular flexibility. In the study, we introduced time differences for displacement, strain, and strain rate changes as indicators of arterial stiffness because of the specificity of this technology. Neither the intima nor adventitia variables were capable of unilateral reflecting the difference in arterial wall movement between the two groups in the present study. In this study, we, for the first time, validated rotation variables, which was not previously possible to assess because only the elasticity average of the intima and media of the carotid artery wall are calculated using other modalities. Interestingly, the time of rotation peak displacement was much earlier in the patient group than in the control group.

Our study has several limitations, including the small number of patients; patients with hypertension, hyperlipidemia, coronary heart disease, and nephropathy were excluded from the study, which limited the number of patients we could enroll. Secondly, although some clinical measurements were different between two groups’ comparisons in the present study, no obvious relationship was found with the arterial stiffness changes by the statistical correlation analysis, further analyze the data with adjustment was not performed as other studies did [28,29]. Thirdly, considering the fact that the number of subjects with the presence of plaque was not sufficient for quantitative statistical analysis, the value of plaque score [30] was not evaluated in this study. Although the patients have significant higher TC, LDL, SBP and DBP than controls, but all these laboratory parameters are still in the normal range. Further research with larger populations should be carried out to verify the present results and validate the changes in arterial stiffness in patients with advanced stage diabetes.

## Conclusions

In conclusion, the present study demonstrated that patients with diabetes have significantly increased arterial stiffness as assessed by QAS and X-strain assessment. QIMT and QAS techniques can be used to noninvasively and comprehensively assess local elastic arterial remodeling in patients with early stage diabetes.

## Abbreviations

CCA: Carotid arterial wall; QIMT: Quality intima-media thickness; QAS: Quality arterial stiffness; PWV: Pulse wave velocity; CC: Compliance coefficient; CVD: Cardiovascular disease; CHD: Coronary heart disease; RF: Radiofrequency; TC: Total cholesterol; TG: Triglycerides; LDL: Low density lipoprotein; HDL: High density lipoprotein; SBP: Systolic blood pressure; DBP: Diastolic blood pressure; SD: Standard deviation; RCCA: Right common carotid artery; BSA: Body surface area; BMI: Body mass index; RD-D: Radial displacement difference; LD-D: Longitudinal displacement difference; ROT-D: Rotation displacement difference; RD-TD: Radial peak displacement; ROT-DT: Time of rotation peak displacement difference; CS-DT: Time of circumferential peak strain difference; RSR-T: Time of radial peak strain rate.

## Competing interests

The authors declare that they have no competing interests.

## Authors’ contributions

LZ devised the study, designed the protocol, participated in fund raising, interpretation of results and prepared the manuscript draft. JKY performed all the statistical analysis. YYD participated in the study design, fund raising and corrected the final version of the manuscript. XL participated in the study design, analytical methods, interpretation of results, and data collection. JW and LX participated in data collection and interpretation of results. YLY participated in the protocol design, fund raising, analysis of results. LJY and TSC participated in the final review of the manuscript. Finally, all authors reviewed and approved the final version of the manuscript.
